# *Cryptococcus* *gattii* in the United States: Genotypic Diversity of Human and Veterinary Isolates

**DOI:** 10.1371/journal.pone.0074737

**Published:** 2013-09-03

**Authors:** Shawn R. Lockhart, Naureen Iqbal, Julie R. Harris, Nina T. Grossman, Emilio DeBess, Ron Wohrle, Nicola Marsden-Haug, Duc J. Vugia

**Affiliations:** 1 Mycotic Diseases Branch, Centers for Disease Control and Prevention, Atlanta, Georgia, United States of America; 2 Oregon Department of Human Services, Portland, Oregon, United States of America; 3 Washington State Department of Health, Tumwater, Washington, United States of America; 4 California Department of Public Health, Richmond, California, United States of America; University of Sydney, Australia

## Abstract

**Background:**

*Cryptococcus* *gattii* infections are being reported in the United States (US) with increasing frequency. Initially, US reports were primarily associated with an ongoing *C* *. gattii* outbreak in the Pacific Northwest (PNW) states of Washington and Oregon, starting in 2004. However, reports of *C* *. gattii* infections in patients from other US states have been increasing since 2009. Whether this is due to increasing frequency of disease, greater recognition within the clinical community, or both is currently unknown.

**Methodology/Principal Findings:**

During 2005–2013, a total of 273 *C* *. gattii* isolates from human and veterinary sources in 16 US states were collected. Of these, 214 (78%) were from the Pacific Northwest (PNW) and comprised primarily the clonal *C* *. gattii* genotypes VGIIa (64%), VGIIc (21%) and VGIIb (9%). The 59 isolates from outside the PNW were predominantly molecular types VGIII (44%) and VGI (41%). Genotyping using multilocus sequence typing revealed small clusters, including a cluster of VGI isolates from the southeastern US, and an unrelated cluster of VGI isolates and a large cluster of VGIII isolates from California. Most of the isolates were mating type MATα, including all of the VGII isolates, but one VGI and three VGIII isolates were mating type MAT**a** .

**Conclusions/Significance:**

We provide the most comprehensive report to date of genotypic diversity of US *C* *. gattii* isolates both inside and outside of the PNW. *C* *. gattii* may have multiple endemic regions in the US, including a previously-unrecognized endemic region in the southeast. Regional clusters exist both in California and the Southeastern US. VGII strains associated with the PNW outbreak do not appear to have spread substantially beyond the PNW.

## Introduction

Cryptococcosis is primarily caused by two species, *Cryptococcus neoformans* and *C* *. gattii* . *Cryptococcus neoformans* is found worldwide and infects both healthy and immunocompromised patients, although in the HIV era it is nearly always identified as a pathogen in immunocompromised hosts [1–3]. *Cryptococcus* *gattii* has a more limited global distribution and, though previously associated with immunocompetent hosts, can cause infection in immunocompromised hosts as well [1,2,4–7]. *Cryptococcus* *gattii* was once thought to be exclusive to tropical and sub-tropical climates, but infections have recently been identified in temperate climates [8–12]. There are four recognized molecular types of *C* *. gattii* : VGI, VGII, VGIII and VGIV [13,14]. The global distribution of these genotypes is not uniform: VGII and VGIII infections are the most-frequently identified isolates in the Americas, VGIV infections occur almost exclusively in Africa, and VGI predominates in Europe, Australia and Asia [5,9,11,15–21].

*Cryptococcus* *gattii* infections have been long recognized as present, but infrequent, in the US [22–26]. By the early 2000s, although sporadic cases had been recorded from a few US states, the only endemic areas in North America were thought to be Southern California and Mexico [7,22–25]. Beginning in 1999, a large number of *C* *. gattii* infections were identified during a short period of time in animals and humans living on temperate Vancouver Island and mainland British Columbia [9,27–30]. Infections were soon identified in the Pacific Northwest (PNW) US as well [31–37]. US cases outside the PNW, especially in California, have been noted in the past [23,26,38–42], but increased awareness of *C* *. gattii* infections associated with the PNW outbreak may have increased awareness of and surveillance for cases from other US states [4,43–48]. Here we report the distribution and genotypic variability of 273 human and veterinary isolates of *C* *. gattii* collected in the United States between January 2005 and January 2013, both inside and outside the PNW region.

## Material and Methods

### Isolate collection

Isolates were submitted to the Centers for Disease Control and Prevention (CDC) through passive surveillance by state public health departments of Oregon and Washington (the disease is reportable in both states), through passive surveillance in other US states, and through requests on various clinical microbiology listserv groups to submit isolates to the CDC Fungus Reference Laboratory. Isolates were confirmed as *C* *. gattii* by melanin production on DOPA media and by the production of a blue color following growth on Canavanine-Glycine-Bromthymol blue media [49]. All isolates were assigned a unique identifier and stored at -80°C.

### Multilocus sequence typing

Isolates were subtyped using multilocus sequence typing (MLST). The *URA5, IGS1, CAP59, LAC1, GPD1, PLB1*, and *SOD1* gene fragments were amplified as described [50] for all isolates. Allele numbers and sequence types (ST) were determined using the online *C* *. gattii* MLST database (http://mlst.mycologylab.org/DefaultInfo.aspx?Page=Cgattii). All new alleles were submitted to the ISHAM MLST database for inclusion and are currently available as part of the database.

### Phylogenetic analysis

All seven MLST alleles for each isolate were concatenated and aligned using the ClustalW function in BioEdit 7.0.0 [51]. Phylogenic analysis was performed using the Mega 4.1 software package [52]. Dendrograms were constructed by using Neighbor-Joining method using the default parameters in the Mega software.

### Mating type determination

Mating type was determined for each isolate by amplification of the MF**a** and MFα pheromones, as described previously [53]. Mating assays were performed on V8 agar as previously described [54] using wild-type strains described in this study as mating pairs.

### Ethics statement

The CDC Human Research Protection Office found this surveillance activity to be exempt from IRB approval as surveillance without identifiers under 45 CFR 46.101(b)(4). No experiments were performed on animals.

## Results

### Strain collection

Between January 2005 and January 2013, the CDC received 169 human isolates and 104 veterinary isolates from 16 US states (Table 1, Table S1). Most isolates were from symptomatic humans or animals living in Oregon (n=151), Washington (n=63), and California (n=32). Twenty-seven (10%) isolates came from other states throughout the continental US, Alaska and Hawaii. Thirteen (48%) of these 27 non-PNW isolates came from the southeast states of South Carolina, Georgia, Florida and Alabama. Sixty-eight (65%) veterinary isolates were from cats and dogs; isolates also came from llamas and alpacas, porpoises and dolphins, goats, elk, horses, sheep, ferrets, and a cow.

**Table 1:** Breakdown of *C* *. gattii* isolates from US passive surveillance by state where isolated.

State	Total	Human	Veterinary*
Oregon	151	88	63
Washington	63	31	32
California	32	28	4
Georgia	8	8	
New Mexico	3	2	1
Idaho	2	1	1
Alabama	2	2	
Hawaii	2	1	1
Florida	2	2	
Michigan	2	2	
Colorado	1		1
Montana	1	1	
Utah	1	1	
Rhode Island	1	1	
Alaska	1		1
South Carolina	1	1	
	273	169	104

^*^ Veterinary = feline (44), canine (24), camelid (11), cetacean (7), caprine (6), cervid (4), equine (3), ovine (2), mustelid (2), bovine (1)

### Molecular typing

The majority of the isolates in this collection (78%) originated in the PNW, and were mostly VGIIa (64%), VGIIb (9%), or VGIIc (21%). A higher proportion of Oregon (n=41, 27%) than Washington (n=3, 5%) isolates was VGIIc (p<0.001), and a lower proportion of Oregon isolates (n=91, 60%) than Washington isolates (n=46, 73%) was VGIIa (p=0.03) (Table 2). For the three patients with VGIIc infection in Washington, acquisition of the infection in neighboring Oregon could not be ruled out. Two isolates from Oregon and one isolate from Washington were identified as molecular type VGII, but not VGIIa, VGIIb or VGIIc. There were three VGII isolates from California, one VGIIa and two VGIIb. Six VGII isolates came from Hawaii, Colorado, Florida, Idaho and Utah. All except the Hawaii and Florida isolates could be associated with a travel history to the PNW.

**Table 2:** Breakdown of *C* *. gattii* genotype by state where isolated.

	Number of isolates					
State	VGI	VGII not a, b or c	VGIIa	VGIIb	VGIIc	VGIII
Oregon	4	2	91	9	41	4
Washington	1	1	46	10	3	2
California	11		1	2		18
Alabama	1					1
New Mexico	1					2
Georgia	6					2
Hawaii	1	1				
Colorado			1			
Florida	1			1		
Idaho		1	1			
Michigan	1					1
Montana	1					
Utah			1			
Rhode Island	1					
Alaska						1
South Carolina						1
	29	5	141	22	44	32

Eleven (5%) isolates from Washington and Oregon were VGI (n=5) or VGIII (n=6). In contrast, 18 (57%) isolates from California were molecular type VGIII and 11 (33%) were VGI. Of the 30 VGIII isolates, 18 (60%) were from California; the rest were from Georgia (n=2), New Mexico (n=2), Alabama (n=1), Michigan (n=1), Alaska (n=1), and South Carolina (n=1). No isolates of molecular type VGIV were identified in this US collection.

**Figure 1:**
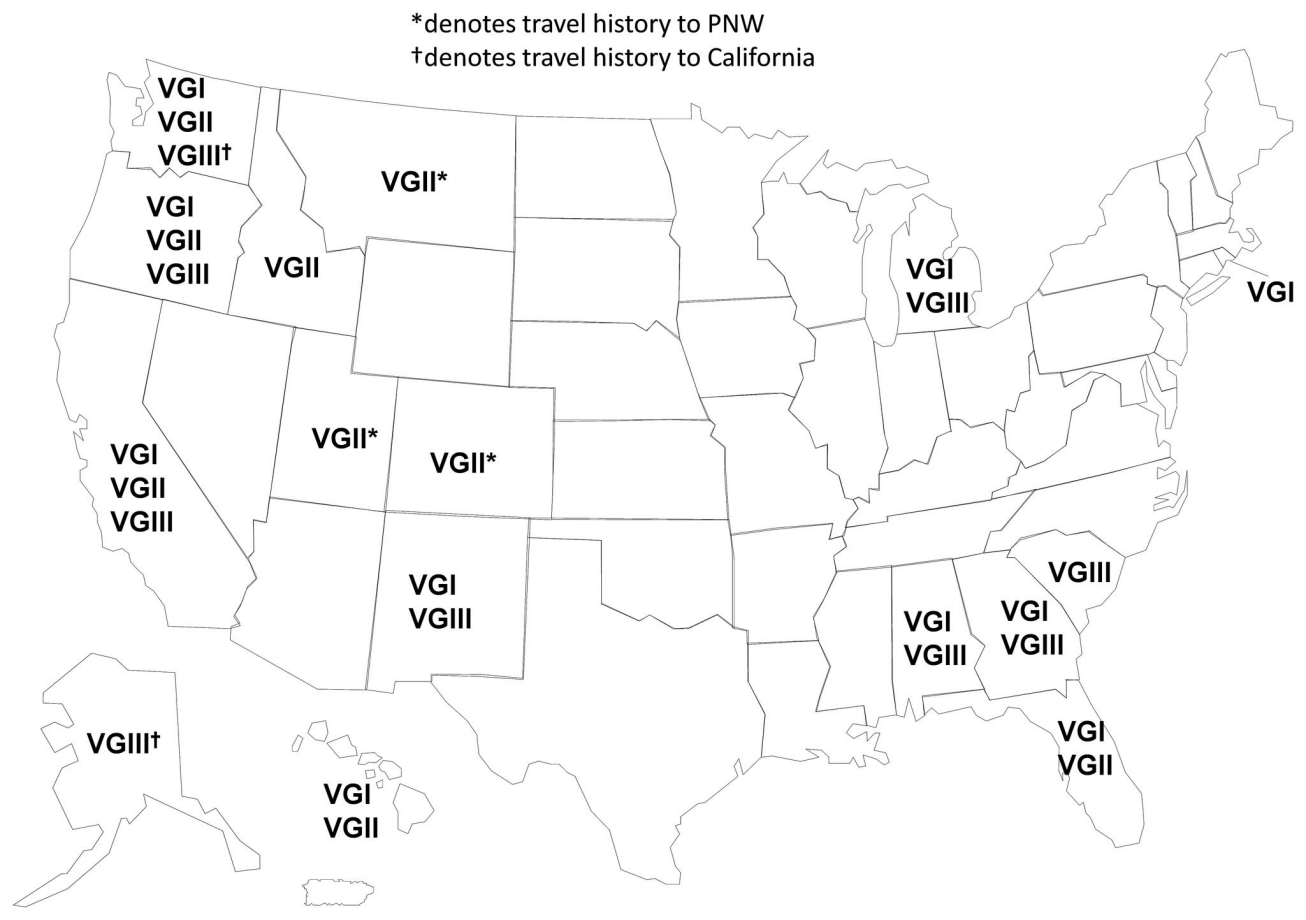
US map showing *C* *. gattii* distribution. The map shows the distribution of *C* *. gattii* molecular types by the state of origin. Because complete travel histories are not known for all patients contributing isolates, it is possible that some infections were acquired in states other than the ones in which they were recognized.

The state with the most *C* *. gattii* isolates outside of the PNW and California was Georgia. Of eight isolates from Georgia, six (75%) were molecular type VGI and two (25%) were molecular type VGIII. Overall, VGI isolates were reported from 11 different states, VGIII isolates were reported from eight states and VGII isolates were reported from eight states (Figure 1).

### Allelic diversity

*LAC 1* was the most informative locus with 17 alleles represented in the collection, 9 of them in VGIII isolates (Table 3). *CAP59* was the least informative locus with only 10 alleles represented. VGIII isolates had the greatest number of total alleles across all loci (n=37) and VGII, despite being the most abundant molecular type by far, had the fewest total alleles across all loci (n=25). There were no alleles that were shared across molecular type; each was unique to VGI, VGII or VGIII.

**Table 3:** Genotypic diversity by allele.

		Genotype		
Allele name	# of alleles	VGI	VGII	VGIII
*GPD1*	13	6	4	5
*CAP59*	10	2	5	4
*PLB1*	12	3	4	5
*LAC1*	17	5	3	9
*SOD1*	15	7	4	4
*URA5*	13	5	2	6
*IGS1*	12	6	3	4
Number of STs^a^		12	7	15

^a^ ST = sequence type

### MLST analysis

Among the 273 isolates, 34 MLST sequence types (STs) were identified. There were 12 STs among the 29 VGI isolates, 15 STs among the 32 VGIII isolates, and seven STs among the 212 VGII isolates. There were only 15 STs among the 214 PNW isolates (VGI had five STs for five isolates; VGII had 5 STs for 203 isolates; VGIII had five STs for six isolates), with approximately equal diversity between human isolates (9 STs) and veterinary isolates (11 STs). Most of the genotypic diversity in the collection originated outside the PNW; there were 24 STs among only 59 isolates. Although the VGI and VGIII isolates were highly diverse, certain STs within each molecular type appeared with greater frequency. Within VGI, nine (31%) of 29 isolates were ST51. These isolates were mostly from California, but also included isolates from Oregon, New Mexico and Rhode Island. The second-most-frequent VGI ST was ST162, comprising five (63%) of eight isolates from Georgia (5/6 VGI isolates from Georgia), one of two from Florida, and one of two from Michigan. Within VGIII, 10 (31%) of 32 isolates were ST75, and three were the closely-related ST139. Most (77%) of these ST75 and ST139 isolates were from California, but one each also came from Oregon, Washington, and Alaska (the Alaska isolate was from a cat that previously resided in California, and the Washington human case was from a person that had recently moved from California to Washington).

### Genotypic distribution among veterinary cases

Among the isolates from dogs, all 24 were VGII isolates (VGIIa=19; VGIIb=3; VGIIc=2). In contrast, in cats, seven (17%) isolates were molecular type VGIII, and one was molecular type VGI. All five isolates from Washington porpoises were VGIIb; however, a single isolate from an Oregon porpoise was VGIIa. All three horse isolates were VGIIb, one each from Oregon, Washington and California. Of the 11 isolates from camelids, eight (72%) were VGIIa, two were VGIIc, and one was VGIII.

### Phylogenetic analysis

**Figure 2:**
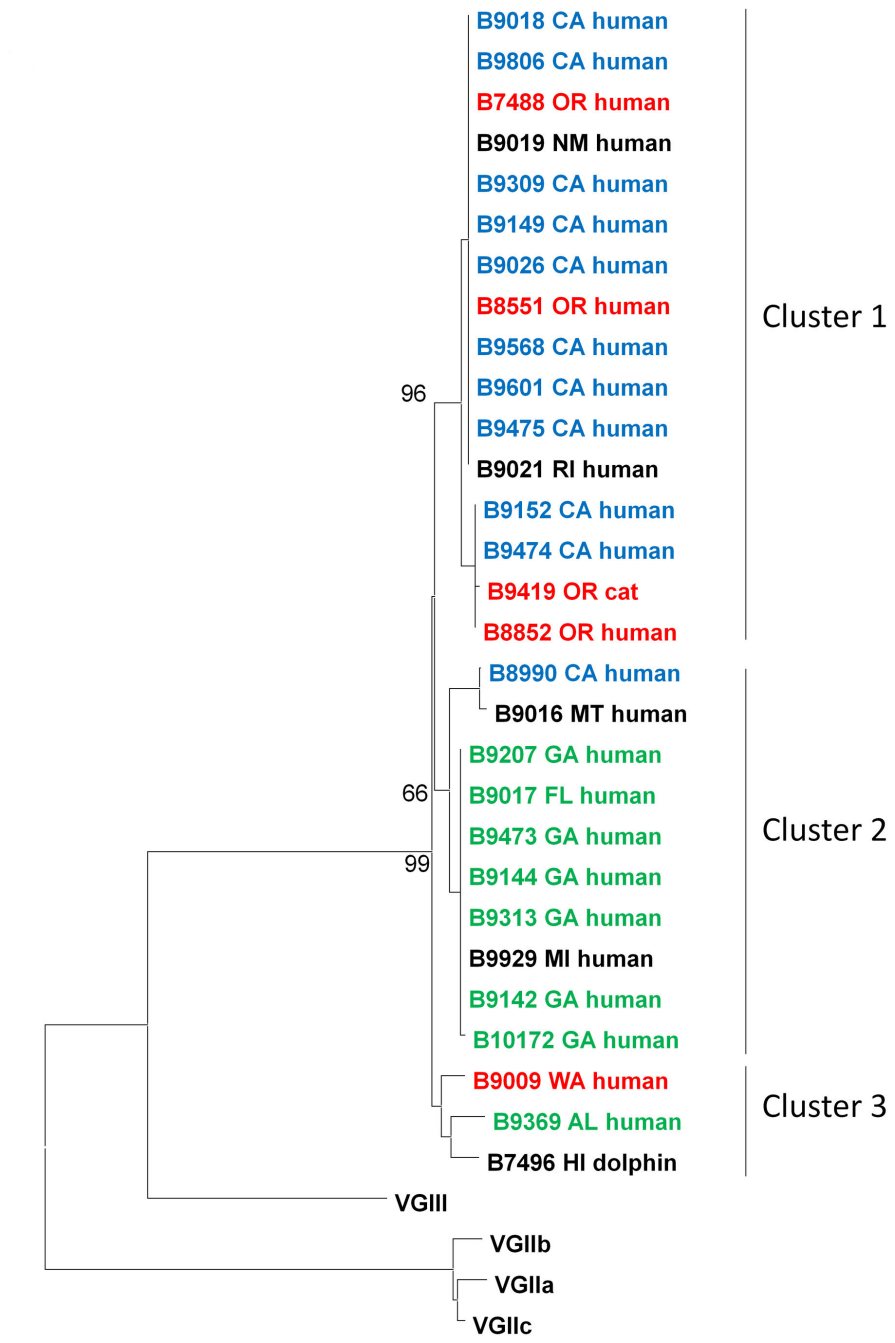
Phylogenetic analysis of VGI isolates. The neighbor-joining tree was constructed using the maximum-likelihood model and reveals three well supported clades among VGI isolates. Bootstrap values (1000 replicates) are shown next to the branches. CA isolates are blue, Pacific Northwest isolates are red and Southeast US isolates are green.

**Figure 3:**
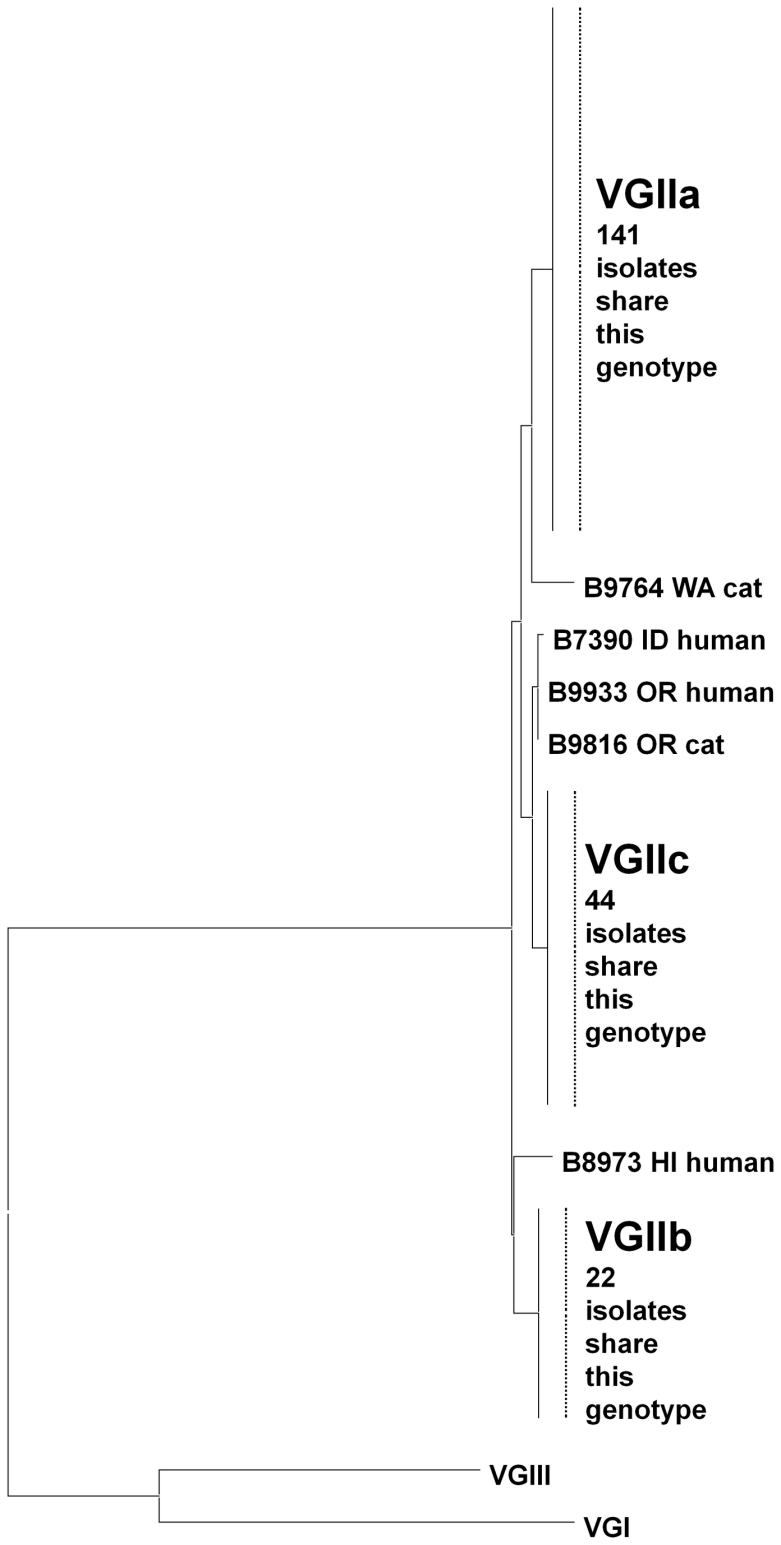
Phylogenetic analysis of VGII isolates. The neighbor-joining tree was constructed using the maximum-likelihood model and reveals that all VGII isolates can be clustered with VGIIa, VGIIb, or VGIIc.

**Figure 4:**
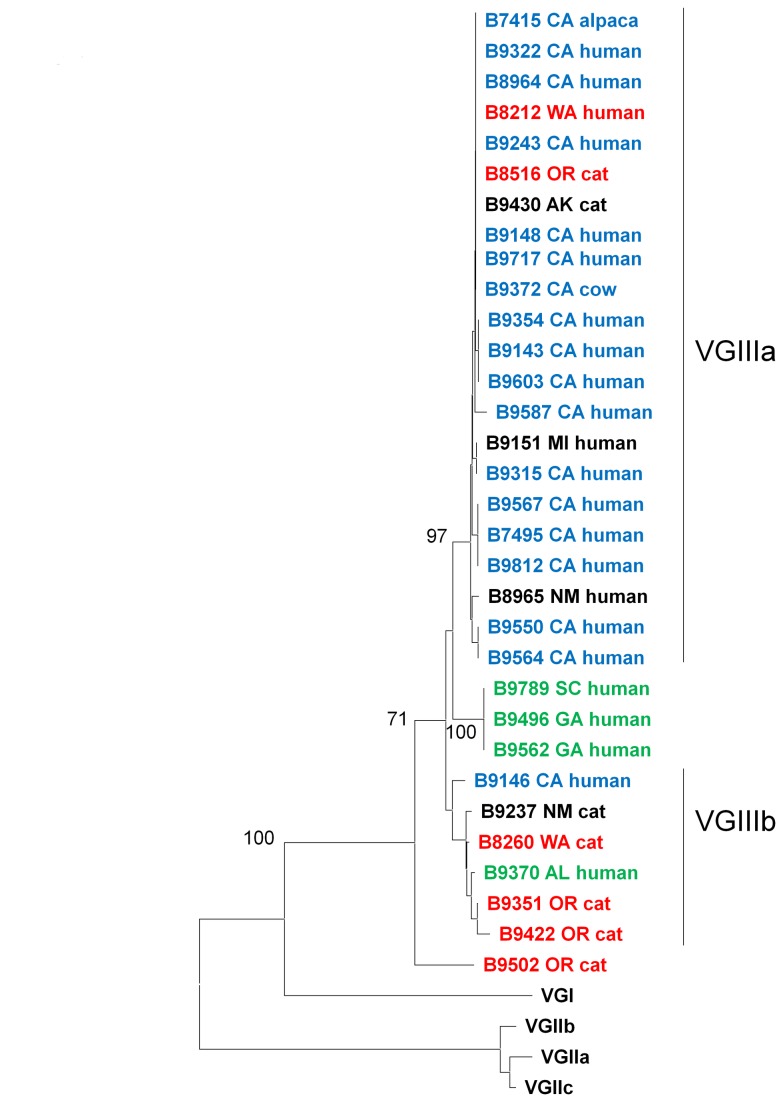
Phylogenetic analysis of VGIII isolates. The neighbor-joining tree was constructed using the maximum-likelihood model and reveals two well supported clades. Bootstrap values (1000 replicates) are shown next to the branches. CA isolates are blue, Pacific Northwest isolates are red and Southeast US isolates are green.

The sequences for all seven loci were concatenated and neighbor-joining trees were derived for each molecular type. VGI isolates could be divided into three clusters with strong bootstrap support (Figure 2). Cluster 1 isolates were primarily from California, while cluster 2 isolates were primarily from the Southeastern US. There were shared alleles between the clades for *CAP59*, *GPD1*, *LAC1*, and *PLB1*. No alleles were shared between clades for *SOD1*, *URA5* and *IGS1*. The VGII isolates were clustered in the major genotypes VGIIa, VGIIb and VGIIc (Figure 3). There were five VGII isolates that were not VGIIa, VGIIb, or VGIIc; three clustered with VGIIc, one clustered with VGIIa and one clustered with VGIIb. Molecular type VGIII isolates could be divided into two clusters with good bootstrap support (Figure 4). Based on common alleles between the two studies, these two lineages correspond to the VGIIIa and VGIIIb lineages that have been previously described for isolates from California [46]. Most VGIIIa isolates originated in California, but the six VGIIIb isolates came from five different states. The isolates from South Carolina and Georgia fell outside of the two clusters. There were shared alleles across the two clusters for *CAP59*, *SOD1*, and *URA5*.

### Mating Type

Mating type (MAT) was determined for all isolates. There were 269 MATα, including all of the VGII isolates, and 4 MAT**a** isolates. The MAT**a** isolates included a VGI isolate from a dolphin from Hawaii, a VGIII isolate from a human case in New Mexico, a VGIII isolate from a human case in California and a VGIII from a cat in Oregon. All four MAT**a** isolates corresponded to a unique ST in the collection. Two of the MAT**a** isolates were confirmed by the production of basidiospores from traditional mating assays with MATα isolates.

## Discussion

This analysis of sequence type diversity of *C* *. gattii* isolates from a US population provides significant new information about this emerging infection in the US. First, the endemic geographic range for *C* *. gattii* in the US extends beyond the PNW and California, and potentially comprises much of the southeast US. Second, *C* *. gattii* genotypic diversity in the US outside of the PNW is much higher than in the PNW. Finally, we identified circulating clusters in the US population that are predominant within each molecular type, and these clusters seem to have geographic associations.

*C. neoformans* has long been divided into four distinct groups by serology [55]. It wasn’t until the 1970s that two of the serotypes, B and C, were recognized as an independent species (species *gattii*) [56–58]. Using serotyping as a retrospective species identification tool, there is a long history of *C* *. gattii* in the US. Wilson and colleagues [39] were the first to recognize that *C. neoformans* isolates from California were primarily serotypes B and C. Shortly after that, Denton and DiSalvo [38] reported a serotype C environmental isolate from an abandoned house in Augusta, Georgia, as well as three patient isolates - two serotype C and one serotype B - from an Augusta Hospital. In 1977, Bennett and colleagues performed serosurveillance on a collection of 272 *Cryptococcus* *spp* isolates from 31 US states [22], reporting 39 serotype B or C isolates from patients in 10 different states, including Alabama, California, Georgia, Minnesota, New Jersey, North Carolina, Oklahoma, Pennsylvania, Texas, and Washington; 25 (64%) came from southern California. Unfortunately, travel histories were not available for patients providing isolates, so possible acquisition in states outside the patients’ state of residence could not be investigated and all environmental isolates within that collection were serotype A or D (species *neoformans*). Fromtling [41] reported isolates of serotype B or C from patients in Alabama, Tennessee, and Louisiana with no travel history to any other endemic region. Older reports document patients from Oklahoma and New York with *C* *. gattii* serotype B and C infections, but past travel to an endemic region could not be ruled out [42,59]. More recently, *C* *. gattii* patients were identified from southern California, Georgia, Rhode Island, New Mexico, Florida, and North Carolina, although the North Carolina patient likely acquired his infection in California [7,26,43–48]. In this study, we identified *C* *. gattii* isolates from 16 different US states. Although travel history was not known for many of the patients, this study indicates that *C* *. gattii* patients are geographically dispersed in the US. Some may have a travel history to endemic regions, but others may have acquired their infection locally in unrecognized endemic regions. Five patients from Georgia, one from Florida, and one from Alabama had no documented travel history outside of the southeast US within the year before their illness, indicating an infection that was likely acquired locally. The tropical climate of HI makes findings of *C* *. gattii* infections there unsurprising. The isolates from Michigan and Montana are perhaps the most intriguing. The Michigan VGIII isolate falls into the clade with VGIII isolates from California (figure 4). The Montana and Michigan VGI isolates fall into the clade with isolates from the southeast US (Figure 2), but it cannot be ruled out that their infections were acquired locally.

Although *C* *. gattii* infections outside of the PNW and California have been documented previously, there has been very little information about the molecular type of the isolates outside of the PNW [43–48]. Here we show that the diversity of *C* *. gattii* genotypes outside of the PNW is high, and that VGI, VGIII, and VGII may all be endemic to the US. Even though the majority of the isolates in this study came from Oregon and Washington, there was less genotypic diversity among the isolates from these two states than among the isolates from the rest of the US with only 15 genotypes identified among 214 isolates from Washington and Oregon; 93% of the isolates from Washington and Oregon were one of three VGII genotypes. This is similar to findings in Australia where genotypic diversity among VGII isolates was low as well, with the majority of isolates in the genotype VGIIb [60]. One possibility is that VGII is the more recent colonizer of North America and Australia and has not yet had a chance to establish clonal diversity in these two geographic locales. It has been predicted that VGII is the ancestral clade of *C* *. gattii* and that it has its origins in South America [14,61]. In support of that hypothesis and in contrast to what was found in the US and Australia, in Brazil the majority of isolates are VGII, but there is a tremendous amount of genotypic diversity among isolates including some closely related to VGIIa and VGIIc (L. Bonfietti and S. Lockhart, unpublished data). We did identify two new VGII STs, one that clustered with VGIIc and one that clustered with VGIIa. Continued surveillance in the PNW will show whether these are single occurrences or new emerging genotypes. The MAT**a** mating type has not been identified among the VGII isolates from the PNW outbreak [35,36], and we did not identify any MAT**a** VGII isolates in this study.

There were 24 genotypes among the 59 isolates collected outside the PNW (one unique ST for every 2.5 isolates). *C* *. gattii* isolates from outside the PNW were almost exclusively molecular types VGI and VGIII. There was significantly more diversity among the VGI and VGIII isolates than among the VGII isolates, with 12 VGI STs and 15 VGIII STs. Consistent with a previous report that identified MAT**a** VGIII isolates in California [46], we found MAT**a** in VGIII isolates from California, New Mexico, and Oregon and a VGI isolate from Hawaii. The presence of isolates of both mating types could lead to more active recombination within the population and could be an explanation for the increased diversity of the VGI and VGIII isolates when compared to the VGII isolates. The increased diversity and the presence of both mating types is also an indicator that VGI and VGIII have been present in the US for much longer than VGII.

Despite the diversity outside of the PNW, there were clusters of STs. There was a cluster of closely related VGI isolates in the southeastern states of Georgia and Florida and a small cluster of VGIII isolates from Georgia and South Carolina. Similar to what had been reported previously from HIV-infected persons [46], this study identified a cluster of closely related VGIII isolates from California, primarily among HIV-uninfected persons [62]. However, we also identified a cluster of VGI isolates from California that was unrelated to the VGI isolates found in the southeastern US. Clusters of a single sequence type spread over a large geographic area may be an indicator of the longevity of a clonal group within the geographic region.

There were several limitations to this study. Surveillance outside of the PNW was passive, and most likely did not include the entire spectrum of *C* *. gattii* disease; that is, mild cases might have been missed, and if different genotypes represent mild versus severe disease, our genetic profile of cases might be biased. Additionally, outside of the PNW, cryptococcal infections are rarely identified to the species level (e.g., *neoformans* versus *gattii*). Second, because limited patient information was collected for many of the cases from outside the PNW, we did not have all of the travel histories and therefore cannot exclude travel-acquired infections for some patients. Finally, veterinary isolates were collected primarily from the PNW and therefore the genotypic diversity that may be present in the veterinary population may be underrepresented in this analysis.

*Cryptococcus* *gattii* is now recognized as endemic to the US. Our results increase knowledge about the diversity of *C* *. gattii* isolates from the US and describe a new endemic region in the Southeastern US. Taking into account historical data from Georgia about *C* *. gattii* , including environmental isolates of *C* *. gattii* from >50 years ago [38], and the 13 cases from Georgia, South Carolina, Florida and Alabama in this report, it seems likely that the southeast US has been an endemic zone for *C* *. gattii* for a long time, with clinical cases perhaps going unrecognized previously.

Further knowledge of the distribution and diversity of *C* *. gattii* in the US is important: recent work has shown that there are differences in antifungal susceptibility between the molecular types [63–65], and there may be differences in clinical presentation between *C* *. gattii* infections with different molecular types [62]. *C* *. gattii* infections often require lengthier treatments and more aggressive interventions [1,2,66–68], making distinction of these infections from *C. neoformans* infections extremely important. Increased vigilance for *C* *. gattii* infection throughout the US will help define the endemic region within the US.

The findings and conclusions of this article are those of the authors and do not necessarily represent the views of the Centers for Disease Control and Prevention.

## Supporting Information

Table S1 **Molecular type origin and sequence type of all isolates analyzed in this study.**
(DOCX)
