# Correction: *Cryptococcus* *gattii* in the United States: Genotypic Diversity of Human and Veterinary Isolates

**DOI:** 10.1371/annotation/c7250cbd-4c85-423a-8b09-da596a72f830

**Published:** 2013-09-12

**Authors:** Shawn R. Lockhart, Naureen Iqbal, Julie R. Harris, Nina T. Grossman, Emilio DeBess, Ron Wohrle, Nicola Marsden-Haug, Duc J. Vugia

The Funding statement should read: his research was supported in part by an appointment to the Emerging Infectious Diseases (EID) Fellowship Program administered by the Association of Public Health Laboratories (APHL) and funded by the Centers for Disease Control and Prevention (CDC). The funders had no role in study design, data collection and analysis, decision to publish, or preparation of the manuscript. 

