# Supplementary material for: Cryptococcus gattii in the United States: Genotypic Diversity of Human and Veterinary Isolates
Source: PLoS One. 2013 Sep 3;8(9):e74737. doi: 10.1371/journal.pone.0074737 (PMC3760847; doi:10.1371/journal.pone.0074737)
Supplement: Table S1 — Molecular type origin and sequence type of all isolates analyzed in this study. (DOCX) [file pone.0074737.s001.docx]

Table S1. Molecular type origin and sequence type of all isolates analyzed in this study

|  |  |  |  | **MLST allele numbers** | | | | | | |  |  |
| --- | --- | --- | --- | --- | --- | --- | --- | --- | --- | --- | --- | --- |
| **Isolate** | **molecular type** | **Origin** | **Source** | **CAP59** | **GPD1** | **LAC1** | **PLB1** | **SOD1** | **URA5** | **IGS1** | **MT** | **ST** |
| B7496 | **VGI** | HI | dolphin | 16 | 33 | 13 | 13 | 47 | 24 | 44 | A | **161** |
| B7488 | **VGI** | OR | human | 16 | 5 | 5 | 5 | 32 | 12 | 3 | alpha | **51** |
| B8551 | **VGI** | OR | human | 16 | 14 | 5 | 5 | 32 | 12 | 3 | alpha | **63** |
| B8852 | **VGI** | OR | human | 16 | 5 | 5 | 5 | 45 | 12 | 3 | alpha | **154** |
| B8990 | **VGI** | CA | human | 16 | 11 | 36 | 5 | 50 | 30 | 47 | alpha | **110** |
| B9009 | **VGI** | WA | human | 36 | 11 | 5 | 13 | 36 | 14 | 13 | alpha | **106** |
| B9016 | **VGI** | MT | human | 16 | 11 | 37 | 5 | 50 | 31 | 48 | alpha | **111** |
| B9017 | **VGI** | FL | human | 16 | 11 | 37 | 5 | 50 | 31 | 47 | alpha | **162** |
| B9018 | **VGI** | CA | human | 16 | 5 | 5 | 5 | 32 | 12 | 3 | alpha | **51** |
| B9019 | **VGI** | NM | human | 16 | 5 | 5 | 5 | 32 | 12 | 3 | alpha | **51** |
| B9021 | **VGI** | RI | human | 16 | 5 | 5 | 5 | 32 | 12 | 3 | alpha | **51** |
| B9026 | **VGI** | CA | human | 16 | 5 | 5 | 5 | 32 | 12 | 3 | alpha | **51** |
| B9142 | **VGI** | GA | human | 16 | 11 | 37 | 5 | 50 | 31 | 47 | alpha | **162** |
| B9144 | **VGI** | GA | human | 16 | 11 | 37 | 5 | 50 | 31 | 47 | alpha | **162** |
| B9149 | **VGI** | CA | human | 16 | 14 | 5 | 5 | 32 | 12 | 3 | alpha | **63** |
| B9152 | **VGI** | CA | human | 16 | 14 | 5 | 5 | 45 | 12 | 3 | alpha | **159** |
| B9207 | **VGI** | GA | human | 16 | 11 | 37 | 5 | 50 | 31 | 47 | alpha | **162** |
| B9309 | **VGI** | CA | human | 16 | 5 | 5 | 5 | 32 | 12 | 3 | alpha | **51** |
| B9313 | **VGI** | GA | human | 16 | 11 | 37 | 5 | 50 | 31 | 47 | alpha | **162** |
| B9369 | **VGI** | AL | human | 16 | 11 | 19 | 15 | 34 | 14 | 13 | alpha | **58** |
| B9419 | **VGI** | OR | cat | 16 | 5 | 5 | 5 | 65 | 12 | 3 | alpha | **57** |
| B9473 | **VGI** | GA | human | 16 | 11 | 37 | 5 | 50 | 31 | 47 | alpha | **162** |
| B9474 | **VGI** | CA | human | 16 | 14 | 5 | 5 | 45 | 12 | 3 | alpha | **159** |
| B9475 | **VGI** | CA | human | 16 | 14 | 5 | 5 | 32 | 12 | 3 | alpha | **63** |
| B9568 | **VGI** | CA | human | 16 | 5 | 5 | 5 | 32 | 12 | 3 | alpha | **51** |
| B9601 | **VGI** | CA | human | 16 | 5 | 5 | 5 | 32 | 12 | 3 | alpha | **51** |
| B9806 | **VGI** | CA | human | 16 | 5 | 5 | 5 | 32 | 12 | 3 | alpha | **51** |
| B9929 | **VGI** | MI | human | 16 | 11 | 37 | 5 | 50 | 31 | 47 | alpha | **162** |
| B10172 | **VGI** | GA | human | 16 | 11 | 37 | 5 | 50 | 31 | 84 | alpha | **269** |
| B7390 | **VGII** | ID | human | 30 | 6 | 4 | 1 | 15 | 2 | 15 | alpha | **49** |
| B8973 | **VGII** | HI | human | 2 | 25 | 21 | 9 | 8 | 7 | 4 | alpha | **50** |
| B9764 | **VGII** | WA | cat | 1 | 17 | 16 | 14 | 19 | 7 | 4 | alpha | **260** |
| B9816 | **VGII** | OR | cat | 4 | 6 | 4 | 1 | 15 | 2 | 15 | alpha | **6** |
| B9933 | **VGII** | OR | human | 4 | 6 | 4 | 1 | 15 | 2 | 15 | alpha | **6** |
| B6864 | **VGIIa** | OR | human | 1 | 1 | 4 | 1 | 14 | 7 | 4 | alpha | **20** |
| B7395 | **VGIIa** | WA | dog | 1 | 1 | 4 | 1 | 14 | 7 | 4 | alpha | **20** |
| B7422 | **VGIIa** | OR | cat | 1 | 1 | 4 | 1 | 14 | 7 | 4 | alpha | **20** |
| B7436 | **VGIIa** | CA | alpaca | 1 | 1 | 4 | 1 | 14 | 7 | 4 | alpha | **20** |
| B7463 | **VGIIa** | OR | dog | 1 | 1 | 4 | 1 | 14 | 7 | 4 | alpha | **20** |
| B7465 | **VGIIa** | OR | camelid | 1 | 1 | 4 | 1 | 14 | 7 | 4 | alpha | **20** |
| B7467 | **VGIIa** | OR | porpoise | 1 | 1 | 4 | 1 | 14 | 7 | 4 | alpha | **20** |
| B7489 | **VGIIa** | OR | human | 1 | 1 | 4 | 1 | 14 | 7 | 4 | alpha | **20** |
| B7490 | **VGIIa** | OR | human | 1 | 1 | 4 | 1 | 14 | 7 | 4 | alpha | **20** |
| B7736 | **VGIIa** | OR | human | 1 | 1 | 4 | 1 | 14 | 7 | 4 | alpha | **20** |
| B8209 | **VGIIa** | OR | human | 1 | 1 | 4 | 1 | 14 | 7 | 4 | alpha | **20** |
| B8217 | **VGIIa** | OR | human | 1 | 1 | 4 | 1 | 14 | 7 | 4 | alpha | **20** |
| B8239 | **VGIIa** | WA | cat | 1 | 1 | 4 | 1 | 14 | 7 | 4 | alpha | **20** |
| B8240 | **VGIIa** | WA | cat | 1 | 1 | 4 | 1 | 14 | 7 | 4 | alpha | **20** |
| B8508 | **VGIIa** | OR | human | 1 | 1 | 4 | 1 | 14 | 7 | 4 | alpha | **20** |
| B8512 | **VGIIa** | OR | alpaca | 1 | 1 | 4 | 1 | 14 | 7 | 4 | alpha | **20** |
| B8517 | **VGIIa** | OR | camelid | 1 | 1 | 4 | 1 | 14 | 7 | 4 | alpha | **20** |
| B8548 | **VGIIa** | OR | human | 1 | 1 | 4 | 1 | 14 | 7 | 4 | alpha | **20** |
| B8550 | **VGIIa** | OR | human | 1 | 1 | 4 | 1 | 14 | 7 | 4 | alpha | **20** |
| B8555 | **VGIIa** | WA | human | 1 | 1 | 4 | 1 | 14 | 7 | 4 | alpha | **20** |
| B8556 | **VGIIa** | WA | human | 1 | 1 | 4 | 1 | 14 | 7 | 4 | alpha | **20** |
| B8557 | **VGIIa** | WA | human | 1 | 1 | 4 | 1 | 14 | 7 | 4 | alpha | **20** |
| B8558 | **VGIIa** | WA | human | 1 | 1 | 4 | 1 | 14 | 7 | 4 | alpha | **20** |
| B8559 | **VGIIa** | WA | human | 1 | 1 | 4 | 1 | 14 | 7 | 4 | alpha | **20** |
| B8560 | **VGIIa** | WA | human | 1 | 1 | 4 | 1 | 14 | 7 | 4 | alpha | **20** |
| B8561 | **VGIIa** | WA | human | 1 | 1 | 4 | 1 | 14 | 7 | 4 | alpha | **20** |
| B8562 | **VGIIa** | WA | human | 1 | 1 | 4 | 1 | 14 | 7 | 4 | alpha | **20** |
| B8563 | **VGIIa** | WA | human | 1 | 1 | 4 | 1 | 14 | 7 | 4 | alpha | **20** |
| B8564 | **VGIIa** | WA | human | 1 | 1 | 4 | 1 | 14 | 7 | 4 | alpha | **20** |
| B8565 | **VGIIa** | WA | human | 1 | 1 | 4 | 1 | 14 | 7 | 4 | alpha | **20** |
| B8566 | **VGIIa** | WA | cat | 1 | 1 | 4 | 1 | 14 | 7 | 4 | alpha | **20** |
| B8567 | **VGIIa** | WA | dog | 1 | 1 | 4 | 1 | 14 | 7 | 4 | alpha | **20** |
| B8570 | **VGIIa** | OR | human | 1 | 1 | 4 | 1 | 14 | 7 | 4 | alpha | **20** |
| B8575 | **VGIIa** | OR | human | 1 | 1 | 4 | 1 | 14 | 7 | 4 | alpha | **20** |
| B8763 | **VGIIa** | OR | human | 1 | 1 | 4 | 1 | 14 | 7 | 4 | alpha | **20** |
| B8764 | **VGIIa** | OR | human | 1 | 1 | 4 | 1 | 14 | 7 | 4 | alpha | **20** |
| B8769 | **VGIIa** | WA | human | 1 | 1 | 4 | 1 | 14 | 7 | 4 | alpha | **20** |
| B8789 | **VGIIa** | OR | human | 1 | 1 | 4 | 1 | 14 | 7 | 4 | alpha | **20** |
| B8792 | **VGIIa** | OR | goat | 1 | 1 | 4 | 1 | 14 | 7 | 4 | alpha | **20** |
| B8793 | **VGIIa** | OR | dog | 1 | 1 | 4 | 1 | 14 | 7 | 4 | alpha | **20** |
| B8794 | **VGIIa** | OR | llama | 1 | 1 | 4 | 1 | 14 | 7 | 4 | alpha | **20** |
| B8796 | **VGIIa** | OR | dog | 1 | 1 | 4 | 1 | 14 | 7 | 4 | alpha | **20** |
| B8797 | **VGIIa** | OR | human | 1 | 1 | 4 | 1 | 14 | 7 | 4 | alpha | **20** |
| B8808 | **VGIIa** | OR | cat | 1 | 1 | 4 | 1 | 14 | 7 | 4 | alpha | **20** |
| B8812 | **VGIIa** | OR | cat | 1 | 1 | 4 | 1 | 14 | 7 | 4 | alpha | **20** |
| B8824 | **VGIIa** | OR | human | 1 | 1 | 4 | 1 | 14 | 7 | 4 | alpha | **20** |
| B8826 | **VGIIa** | OR | human | 1 | 1 | 4 | 1 | 14 | 7 | 4 | alpha | **20** |
| B8834 | **VGIIa** | OR | elk | 1 | 1 | 4 | 1 | 14 | 7 | 4 | alpha | **20** |
| B8839 | **VGIIa** | WA | cat | 1 | 1 | 4 | 1 | 14 | 7 | 4 | alpha | **20** |
| B8840 | **VGIIa** | WA | dog | 1 | 1 | 4 | 1 | 14 | 7 | 4 | alpha | **20** |
| B8845 | **VGIIa** | WA | cat | 1 | 1 | 4 | 1 | 14 | 7 | 4 | alpha | **20** |
| B8846 | **VGIIa** | OR | human | 1 | 1 | 4 | 1 | 14 | 7 | 4 | alpha | **20** |
| B8847 | **VGIIa** | OR | human | 1 | 1 | 4 | 1 | 14 | 7 | 4 | alpha | **20** |
| B8854 | **VGIIa** | WA | human | 1 | 1 | 4 | 1 | 14 | 7 | 4 | alpha | **20** |
| B8858 | **VGIIa** | OR | dog | 1 | 1 | 4 | 1 | 14 | 7 | 4 | alpha | **20** |
| B8918 | **VGIIa** | WA | human | 1 | 1 | 4 | 1 | 14 | 7 | 4 | alpha | **20** |
| B8929 | **VGIIa** | OR | human | 1 | 1 | 4 | 1 | 14 | 7 | 4 | alpha | **20** |
| B8955 | **VGIIa** | OR | goat | 1 | 1 | 4 | 1 | 14 | 7 | 4 | alpha | **20** |
| B8970 | **VGIIa** | OR | human | 1 | 1 | 4 | 1 | 14 | 7 | 4 | alpha | **20** |
| B8979 | **VGIIa** | OR | human | 1 | 1 | 4 | 1 | 14 | 7 | 4 | alpha | **20** |
| B8980 | **VGIIa** | OR | human | 1 | 1 | 4 | 1 | 14 | 7 | 4 | alpha | **20** |
| B8981 | **VGIIa** | OR | human | 1 | 1 | 4 | 1 | 14 | 7 | 4 | alpha | **20** |
| B8982 | **VGIIa** | OR | human | 1 | 1 | 4 | 1 | 14 | 7 | 4 | alpha | **20** |
| B9022 | **VGIIa** | OR | human | 1 | 1 | 4 | 1 | 14 | 7 | 4 | alpha | **20** |
| B9023 | **VGIIa** | OR | human | 1 | 1 | 4 | 1 | 14 | 7 | 4 | alpha | **20** |
| B9087 | **VGIIa** | OR | human | 1 | 1 | 4 | 1 | 14 | 7 | 4 | alpha | **20** |
| B9141 | **VGIIa** | UT | human | 1 | 1 | 4 | 1 | 14 | 7 | 4 | alpha | **20** |
| B9150 | **VGIIa** | WA | human | 1 | 1 | 4 | 1 | 14 | 7 | 4 | alpha | **20** |
| B9156 | **VGIIa** | CO | camelid | 1 | 1 | 4 | 1 | 14 | 7 | 4 | alpha | **20** |
| B9158 | **VGIIa** | WA | dog | 1 | 1 | 4 | 1 | 14 | 7 | 4 | alpha | **20** |
| B9165 | **VGIIa** | OR | human | 1 | 1 | 4 | 1 | 14 | 7 | 4 | alpha | **20** |
| B9167 | **VGIIa** | OR | human | 1 | 1 | 4 | 1 | 14 | 7 | 4 | alpha | **20** |
| B9175 | **VGIIa** | OR | dog | 1 | 1 | 4 | 1 | 14 | 7 | 4 | alpha | **20** |
| B9176 | **VGIIa** | WA | human | 1 | 1 | 4 | 1 | 14 | 7 | 4 | alpha | **20** |
| B9184 | **VGIIa** | WA | dog | 1 | 1 | 4 | 1 | 14 | 7 | 4 | alpha | **20** |
| B9187 | **VGIIa** | WA | elk | 1 | 1 | 4 | 1 | 14 | 7 | 4 | alpha | **20** |
| B9201 | **VGIIa** | WA | human | 1 | 1 | 4 | 1 | 14 | 7 | 4 | alpha | **20** |
| B9204 | **VGIIa** | OR | human | 1 | 1 | 4 | 1 | 14 | 7 | 4 | alpha | **20** |
| B9205 | **VGIIa** | OR | goat | 1 | 1 | 4 | 1 | 14 | 7 | 4 | alpha | **20** |
| B9226 | **VGIIa** | OR | cat | 1 | 1 | 4 | 1 | 14 | 7 | 4 | alpha | **20** |
| B9233 | **VGIIa** | WA | human | 1 | 1 | 4 | 1 | 14 | 7 | 4 | alpha | **20** |
| B9240 | **VGIIa** | OR | human | 1 | 1 | 4 | 1 | 14 | 7 | 4 | alpha | **20** |
| B9241 | **VGIIa** | OR | human | 1 | 1 | 4 | 1 | 14 | 7 | 4 | alpha | **20** |
| B9242 | **VGIIa** | OR | human | 1 | 1 | 4 | 1 | 14 | 7 | 4 | alpha | **20** |
| B9248 | **VGIIa** | OR | ferret | 1 | 1 | 4 | 1 | 14 | 7 | 4 | alpha | **20** |
| B9294 | **VGIIa** | OR | human | 1 | 1 | 4 | 1 | 14 | 7 | 4 | alpha | **20** |
| B9317 | **VGIIa** | OR | camelid | 1 | 1 | 4 | 1 | 14 | 7 | 4 | alpha | **20** |
| B9320 | **VGIIa** | OR | cat | 1 | 1 | 4 | 1 | 14 | 7 | 4 | alpha | **20** |
| B9352 | **VGIIa** | OR | human | 1 | 1 | 4 | 1 | 14 | 7 | 4 | alpha | **20** |
| B9353 | **VGIIa** | WA | cat | 1 | 1 | 4 | 1 | 14 | 7 | 4 | alpha | **20** |
| B9358 | **VGIIa** | OR | goat | 1 | 1 | 4 | 1 | 14 | 7 | 4 | alpha | **20** |
| B9363 | **VGIIa** | OR | human | 1 | 1 | 4 | 1 | 14 | 7 | 4 | alpha | **20** |
| B9379 | **VGIIa** | WA | cat | 1 | 1 | 4 | 1 | 14 | 7 | 4 | alpha | **20** |
| B9390 | **VGIIa** | WA | cat | 1 | 1 | 4 | 1 | 14 | 7 | 4 | alpha | **20** |
| B9416 | **VGIIa** | WA | cat | 1 | 1 | 4 | 1 | 14 | 7 | 4 | alpha | **20** |
| B9418 | **VGIIa** | ID | dog | 1 | 1 | 4 | 1 | 14 | 7 | 4 | alpha | **20** |
| B9426 | **VGIIa** | OR | camelid | 1 | 1 | 4 | 1 | 14 | 7 | 4 | alpha | **20** |
| B9427 | **VGIIa** | OR | human | 1 | 1 | 4 | 1 | 14 | 7 | 4 | alpha | **20** |
| B9431 | **VGIIa** | OR | cat | 1 | 1 | 4 | 1 | 14 | 7 | 4 | alpha | **20** |
| B9434 | **VGIIa** | WA | human | 1 | 1 | 4 | 1 | 14 | 7 | 4 | alpha | **20** |
| B9456 | **VGIIa** | OR | elk | 1 | 1 | 4 | 1 | 14 | 7 | 4 | alpha | **20** |
| B9457 | **VGIIa** | OR | human | 1 | 1 | 4 | 1 | 14 | 7 | 4 | alpha | **20** |
| B9459 | **VGIIa** | OR | human | 1 | 1 | 4 | 1 | 14 | 7 | 4 | alpha | **20** |
| B9462 | **VGIIa** | OR | cat | 1 | 1 | 4 | 1 | 14 | 7 | 4 | alpha | **20** |
| B10175 | **VGIIa** | WA | human | 1 | 1 | 4 | 1 | 14 | 7 | 4 | alpha | **20** |
| B10181 | **VGIIa** | OR | human | 1 | 1 | 4 | 1 | 14 | 7 | 4 | alpha | **20** |
| B10189 | **VGIIa** | WA | human | 1 | 1 | 4 | 1 | 14 | 7 | 4 | alpha | **20** |
| B9480 | **VGIIa** | OR | human | 1 | 6 | 4 | 1 | 14 | 7 | 4 | alpha | **20** |
| B9483 | **VGIIa** | OR | human | 1 | 1 | 4 | 1 | 14 | 7 | 4 | alpha | **20** |
| B9503 | **VGIIa** | OR | dog | 1 | 1 | 4 | 1 | 14 | 7 | 4 | alpha | **20** |
| B9506 | **VGIIa** | OR | human | 1 | 1 | 4 | 1 | 14 | 7 | 4 | alpha | **20** |
| B9512 | **VGIIa** | OR | dog | 1 | 1 | 4 | 1 | 14 | 7 | 4 | alpha | **20** |
| B9546 | **VGIIa** | WA | dog | 1 | 1 | 4 | 1 | 14 | 7 | 4 | alpha | **20** |
| B9547 | **VGIIa** | WA | dog | 1 | 1 | 4 | 1 | 14 | 7 | 4 | alpha | **20** |
| B9548 | **VGIIa** | WA | human | 1 | 1 | 4 | 1 | 14 | 7 | 4 | alpha | **20** |
| B9551 | **VGIIa** | OR | cat | 1 | 1 | 4 | 1 | 14 | 7 | 4 | alpha | **20** |
| B9553 | **VGIIa** | OR | human | 1 | 1 | 4 | 1 | 14 | 7 | 4 | alpha | **20** |
| B9565 | **VGIIa** | WA | dog | 1 | 1 | 4 | 1 | 14 | 7 | 4 | alpha | **20** |
| B9583 | **VGIIa** | OR | dog | 1 | 1 | 4 | 1 | 14 | 7 | 4 | alpha | **20** |
| B9584 | **VGIIa** | OR | cat | 1 | 1 | 4 | 1 | 14 | 7 | 4 | alpha | **20** |
| B9585 | **VGIIa** | WA | human | 1 | 1 | 4 | 1 | 14 | 7 | 4 | alpha | **20** |
| B9600 | **VGIIa** | OR | cat | 1 | 1 | 4 | 1 | 14 | 7 | 4 | alpha | **20** |
| B9602 | **VGIIa** | OR | dog | 1 | 1 | 4 | 1 | 14 | 7 | 4 | alpha | **20** |
| B9701 | **VGIIa** | OR | human | 1 | 1 | 4 | 1 | 14 | 7 | 4 | alpha | **20** |
| B9719 | **VGIIa** | OR | human | 1 | 1 | 4 | 1 | 14 | 7 | 4 | alpha | **20** |
| B9721 | **VGIIa** | WA | cat | 1 | 1 | 4 | 1 | 14 | 7 | 4 | alpha | **20** |
| B9725 | **VGIIa** | WA | human | 1 | 1 | 4 | 1 | 14 | 7 | 4 | alpha | **20** |
| B9726 | **VGIIa** | WA | human | 1 | 1 | 4 | 1 | 14 | 7 | 4 | alpha | **20** |
| B9750 | **VGIIa** | OR | ferret | 1 | 1 | 4 | 1 | 14 | 7 | 4 | alpha | **20** |
| B9754 | **VGIIa** | OR | human | 1 | 1 | 4 | 1 | 14 | 7 | 4 | alpha | **20** |
| B9763 | **VGIIa** | OR | dog | 1 | 1 | 4 | 1 | 14 | 7 | 4 | alpha | **20** |
| B9783 | **VGIIa** | WA | human | 1 | 1 | 4 | 1 | 14 | 7 | 4 | alpha | **20** |
| B9786 | **VGIIa** | OR | human | 1 | 1 | 4 | 1 | 14 | 7 | 4 | alpha | **20** |
| B9787 | **VGIIa** | OR | goat | 1 | 1 | 4 | 1 | 14 | 7 | 4 | alpha | **20** |
| B9790 | **VGIIa** | WA | human | 1 | 1 | 4 | 1 | 14 | 7 | 4 | alpha | **20** |
| B9809 | **VGIIa** | OR | human | 1 | 1 | 4 | 1 | 14 | 7 | 4 | alpha | **20** |
| B9813 | **VGIIa** | OR | goat | 1 | 1 | 4 | 1 | 14 | 7 | 4 | alpha | **20** |
| B9815 | **VGIIa** | OR | cat | 1 | 1 | 4 | 1 | 14 | 7 | 4 | alpha | **20** |
| B9748 | **VGIIa** | OR | human | 1 | 1 | 4 | 1 | 14 | 7 | 4 | alpha | **20** |
| B9749 | **VGIIa** | OR | human | 1 | 1 | 4 | 1 | 14 | 7 | 4 | alpha | **20** |
| B10164 | **VGIIa** | OR | human | 1 | 1 | 4 | 1 | 14 | 7 | 4 | alpha | **20** |
| B7394 | **VGIIb** | WA | cat | 2 | 6 | 4 | 2 | 15 | 2 | 10 | alpha | **7** |
| B7414 | **VGIIb** | CA | horse | 2 | 6 | 4 | 2 | 15 | 2 | 10 | alpha | **7** |
| B7435 | **VGIIb** | OR | elk | 2 | 6 | 4 | 2 | 15 | 2 | 10 | alpha | **7** |
| B7735 | **VGIIb** | OR | human | 2 | 6 | 4 | 2 | 15 | 2 | 10 | alpha | **7** |
| B8211 | **VGIIb** | OR | human | 2 | 6 | 4 | 2 | 15 | 2 | 10 | alpha | **7** |
| B8216 | **VGIIb** | OR | cat | 2 | 6 | 4 | 2 | 15 | 2 | 10 | alpha | **7** |
| B8547 | **VGIIb** | OR | human | 2 | 6 | 4 | 2 | 15 | 2 | 10 | alpha | **7** |
| B8554 | **VGIIb** | OR | dog | 2 | 6 | 4 | 2 | 15 | 2 | 10 | alpha | **7** |
| B8828 | **VGIIb** | WA | porpoise | 2 | 6 | 4 | 2 | 15 | 2 | 10 | alpha | **7** |
| B8966 | **VGIIb** | OR | horse | 2 | 6 | 4 | 2 | 15 | 2 | 10 | alpha | **7** |
| B9157 | **VGIIb** | WA | horse | 2 | 6 | 4 | 2 | 15 | 2 | 10 | alpha | **7** |
| B9168 | **VGIIb** | WA | porpoise | 2 | 6 | 4 | 2 | 15 | 2 | 10 | alpha | **7** |
| B9234 | **VGIIb** | WA | cat | 2 | 6 | 4 | 2 | 15 | 2 | 10 | alpha | **7** |
| B9359 | **VGIIb** | CA | human | 2 | 6 | 4 | 2 | 15 | 2 | 10 | alpha | **7** |
| B9360 | **VGIIb** | WA | porpoise | 2 | 6 | 4 | 2 | 15 | 2 | 10 | alpha | **7** |
| B9421 | **VGIIb** | WA | dog | 2 | 6 | 4 | 2 | 15 | 2 | 10 | alpha | **7** |
| B9428 | **VGIIb** | WA | cat | 2 | 6 | 4 | 2 | 15 | 2 | 10 | alpha | **7** |
| B9469 | **VGIIb** | OR | dog | 2 | 6 | 4 | 2 | 15 | 2 | 10 | alpha | **7** |
| B9563 | **VGIIb** | WA | porpoise | 2 | 6 | 4 | 2 | 15 | 2 | 10 | alpha | **7** |
| B9552 | **VGIIb** | WA | porpoise | 2 | 6 | 4 | 2 | 15 | 2 | 10 | alpha | **7** |
| B9588 | **VGIIb** | FL | human | 2 | 6 | 4 | 2 | 15 | 2 | 10 | alpha | **7** |
| B9788 | **VGIIb** | OR | cat | 2 | 6 | 4 | 2 | 15 | 2 | 10 | alpha | **7** |
| B7432 | **VGIIc** | OR | human | 6 | 6 | 4 | 1 | 15 | 2 | 15 | alpha | **88** |
| B7434 | **VGIIc** | OR | human | 6 | 6 | 4 | 1 | 15 | 2 | 15 | alpha | **88** |
| B7466 | **VGIIc** | OR | cat | 6 | 6 | 4 | 1 | 15 | 2 | 15 | alpha | **88** |
| B7491 | **VGIIc** | OR | human | 6 | 6 | 4 | 1 | 15 | 2 | 15 | alpha | **88** |
| B7493 | **VGIIc** | OR | sheep | 6 | 6 | 4 | 1 | 15 | 2 | 15 | alpha | **88** |
| B7641 | **VGIIc** | OR | cat | 6 | 6 | 4 | 1 | 15 | 2 | 15 | alpha | **88** |
| B7737 | **VGIIc** | OR | human | 6 | 6 | 4 | 1 | 15 | 2 | 15 | alpha | **88** |
| B7765 | **VGIIc** | OR | dog | 6 | 6 | 4 | 1 | 15 | 2 | 15 | alpha | **88** |
| B8210 | **VGIIc** | OR | human | 6 | 6 | 4 | 1 | 15 | 2 | 15 | alpha | **88** |
| B8214 | **VGIIc** | OR | human | 6 | 6 | 4 | 1 | 15 | 2 | 15 | alpha | **88** |
| B8510 | **VGIIc** | OR | human | 6 | 6 | 4 | 1 | 15 | 2 | 15 | alpha | **88** |
| B8542 | **VGIIc** | OR | human | 6 | 6 | 4 | 1 | 15 | 2 | 15 | alpha | **88** |
| B8543 | **VGIIc** | OR | human | 6 | 6 | 4 | 1 | 15 | 2 | 15 | alpha | **88** |
| B8549 | **VGIIc** | OR | human | 6 | 6 | 4 | 1 | 15 | 2 | 15 | alpha | **88** |
| B8552 | **VGIIc** | OR | human | 6 | 6 | 4 | 1 | 15 | 2 | 15 | alpha | **88** |
| B8571 | **VGIIc** | WA | human | 6 | 6 | 4 | 1 | 15 | 2 | 15 | alpha | **88** |
| B8788 | **VGIIc** | OR | human | 6 | 6 | 4 | 1 | 15 | 2 | 15 | alpha | **88** |
| B8798 | **VGIIc** | OR | human | 6 | 6 | 4 | 1 | 15 | 2 | 15 | alpha | **88** |
| B8821 | **VGIIc** | OR | human | 6 | 6 | 4 | 1 | 15 | 2 | 15 | alpha | **88** |
| B8825 | **VGIIc** | OR | human | 6 | 6 | 4 | 1 | 15 | 2 | 15 | alpha | **88** |
| B8833 | **VGIIc** | OR | cat | 6 | 6 | 4 | 1 | 15 | 2 | 15 | alpha | **88** |
| B8838 | **VGIIc** | WA | human | 6 | 6 | 4 | 1 | 15 | 2 | 15 | alpha | **88** |
| B8843 | **VGIIc** | OR | human | 6 | 6 | 4 | 1 | 15 | 2 | 15 | alpha | **88** |
| B8853 | **VGIIc** | OR | cat | 6 | 6 | 4 | 1 | 15 | 2 | 15 | alpha | **88** |
| B9159 | **VGIIc** | WA | sheep | 6 | 6 | 4 | 1 | 15 | 2 | 15 | alpha | **88** |
| B9171 | **VGIIc** | OR | dog | 6 | 6 | 4 | 1 | 15 | 2 | 15 | alpha | **88** |
| B9227 | **VGIIc** | OR | cat | 6 | 6 | 4 | 1 | 15 | 2 | 15 | alpha | **88** |
| B9235 | **VGIIc** | OR | human | 6 | 6 | 4 | 1 | 15 | 2 | 15 | alpha | **88** |
| B9244 | **VGIIc** | OR | human | 6 | 6 | 4 | 1 | 15 | 2 | 15 | alpha | **88** |
| B9245 | **VGIIc** | OR | human | 6 | 6 | 4 | 1 | 15 | 2 | 15 | alpha | **88** |
| B9373 | **VGIIc** | OR | human | 6 | 6 | 4 | 1 | 15 | 2 | 15 | alpha | **88** |
| B9374 | **VGIIc** | OR | human | 6 | 6 | 4 | 1 | 15 | 2 | 15 | alpha | **88** |
| B9380 | **VGIIc** | OR | cat | 6 | 6 | 4 | 1 | 15 | 2 | 15 | alpha | **88** |
| B9477 | **VGIIc** | OR | human | 6 | 6 | 4 | 1 | 15 | 2 | 15 | alpha | **88** |
| B9478 | **VGIIc** | OR | human | 6 | 6 | 4 | 1 | 15 | 2 | 15 | alpha | **88** |
| B6863 | **VGIIc** | OR | human | 6 | 6 | 4 | 1 | 15 | 2 | 15 | alpha | **88** |
| B9495 | **VGIIc** | OR | cat | 6 | 6 | 4 | 1 | 15 | 2 | 15 | alpha | **88** |
| B9501 | **VGIIc** | OR | camelid | 6 | 6 | 4 | 1 | 15 | 2 | 15 | alpha | **88** |
| B9558 | **VGIIc** | OR | human | 6 | 6 | 4 | 1 | 15 | 2 | 15 | alpha | **88** |
| B9566 | **VGIIc** | OR | human | 6 | 6 | 4 | 1 | 15 | 2 | 15 | alpha | **88** |
| B9582 | **VGIIc** | OR | cat | 6 | 6 | 4 | 1 | 15 | 2 | 15 | alpha | **88** |
| B9697 | **VGIIc** | OR | human | 6 | 6 | 4 | 1 | 15 | 2 | 15 | alpha | **88** |
| B9727 | **VGIIc** | OR | alpaca | 6 | 6 | 4 | 1 | 15 | 2 | 15 | alpha | **88** |
| B9755 | **VGIIc** | OR | human | 6 | 6 | 4 | 1 | 15 | 2 | 15 | alpha | **88** |
| B8965 | **VGIII** | NM | human | 18 | 3 | 3 | 20 | 28 | 25 | 1 | A | **112** |
| B9146 | **VGIII** | CA | human | 18 | 12 | 14 | 4 | 28 | 26 | 5 | A | **140** |
| B9502 | **VGIII** | OR | cat | 29 | 7 | 9 | 23 | 66 | 21 | 18 | A | **163** |
| B7415 | **VGIII** | CA | alpaca | 18 | 3 | 3 | 6 | 40 | 19 | 1 | alpha | **75** |
| B7495 | **VGIII** | CA | human | 18 | 3 | 32 | 20 | 40 | 23 | 1 | alpha | **74** |
| B8212 | **VGIII** | WA | human | 18 | 3 | 3 | 6 | 40 | 19 | 1 | alpha | **75** |
| B8260 | **VGIII** | WA | cat | 29 | 9 | 2 | 4 | 28 | 19 | 18 | alpha | **61** |
| B8516 | **VGIII** | OR | cat | 18 | 3 | 3 | 6 | 40 | 19 | 1 | alpha | **75** |
| B8964 | **VGIII** | CA | human | 18 | 3 | 3 | 6 | 40 | 19 | 1 | alpha | **75** |
| B9143 | **VGIII** | CA | human | 18 | 3 | 15 | 6 | 40 | 19 | 1 | alpha | **139** |
| B9148 | **VGIII** | CA | human | 18 | 3 | 3 | 6 | 40 | 19 | 1 | alpha | **75** |
| B9151 | **VGIII** | MI | human | 18 | 3 | 3 | 17 | 40 | 19 | 1 | alpha | **138** |
| B9237 | **VGIII** | NM | cat | 29 | 7 | 39 | 4 | 28 | 19 | 5 | alpha | **141** |
| B9243 | **VGIII** | CA | human | 18 | 3 | 3 | 6 | 40 | 19 | 1 | alpha | **75** |
| B9315 | **VGIII** | CA | human | 18 | 3 | 3 | 17 | 40 | 19 | 1 | alpha | **138** |
| B9322 | **VGIII** | CA | human | 18 | 3 | 3 | 6 | 40 | 19 | 1 | alpha | **75** |
| B9351 | **VGIII** | OR | cat | 29 | 9 | 2 | 4 | 28 | 21 | 11 | alpha | **142** |
| B9354 | **VGIII** | CA | human | 18 | 3 | 15 | 6 | 40 | 19 | 1 | alpha | **139** |
| B9370 | **VGIII** | AL | human | 29 | 7 | 2 | 4 | 28 | 21 | 5 | alpha | **79** |
| B9372 | **VGIII** | CA | cow | 18 | 3 | 3 | 6 | 40 | 19 | 1 | alpha | **75** |
| B9422 | **VGIII** | OR | cat | 29 | 12 | 9 | 4 | 28 | 22 | 11 | alpha | **164** |
| B9430 | **VGIII** | AK | cat | 18 | 3 | 3 | 6 | 40 | 19 | 1 | alpha | **75** |
| B9496 | **VGIII** | GA | human | 35 | 40 | 22 | 6 | 28 | 19 | 1 | alpha | **165** |
| B9550 | **VGIII** | CA | human | 18 | 3 | 32 | 20 | 38 | 19 | 1 | alpha | **113** |
| B9562 | **VGIII** | GA | human | 35 | 40 | 22 | 6 | 28 | 19 | 1 | alpha | **165** |
| B9564 | **VGIII** | CA | human | 18 | 3 | 32 | 20 | 38 | 19 | 1 | alpha | **113** |
| B9567 | **VGIII** | CA | human | 18 | 3 | 32 | 20 | 40 | 23 | 1 | alpha | **74** |
| B9587 | **VGIII** | CA | human | 46 | 3 | 35 | 6 | 40 | 23 | 1 | alpha | **166** |
| B9603 | **VGIII** | CA | human | 18 | 3 | 15 | 6 | 40 | 19 | 1 | alpha | **139** |
| B9717 | **VGIII** | CA | human | 18 | 3 | 3 | 6 | 40 | 19 | 1 | alpha | **75** |
| B9789 | **VGIII** | SC | human | 35 | 40 | 22 | 6 | 28 | 19 | 1 | alpha | **165** |
| B9812 | **VGIII** | CA | human | 18 | 3 | 32 | 20 | 40 | 23 | 1 | alpha | **74** |

MT-mating type; ST-sequence type; AK-Alaska; AL-Alabama; CA-California; CO-Colorado; FL-Florida; GA-Georgia; HI-Hawaii; ID-Idaho; MI-Michigan; MT-Montana; NM-New Mexico; OR-Oregon; RI-Rhode Island; SC-South Carolina; UT-Utah; WA-Washington
